# In-bore climate control chamber for magnetic resonance imaging of living plants

**DOI:** 10.1186/s13007-026-01561-2

**Published:** 2026-07-07

**Authors:** N. Knorr, P. Rovedo, J. Leupold, D. von Elverfeldt, L. Hesse, S. Bär

**Affiliations:** 1 Biomimetics Group, Institute of Wood Science, Department of Biology, University of Hamburg, Hamburg, Germany; 2 Division of Medical Physics, Department of Diagnostic and Interventional Radiology, University Medical Center Freiburg, Faculty of Medicine, University of Freiburg, Freiburg, Germany; 3 Neurocenter, Medical Center, Faculty of Medicine, University of Freiburg, Freiburg, Germany

**Keywords:** Plant MRI, Functional imaging, Climate control, Open-source hardware, Sap flow velocity, Climate control, Functional imaging, MRI, Low cost, Open source, Bio-imaging

## Abstract

Magnetic resonance imaging (MRI) enables non-invasive and non-destructive, three-dimensional anatomical and functional imaging of plant tissues and the quantitative investigation of dynamic processes such as water transport. Despite these advantages, MRI remains underutilized in plant and biomimetic research. One major limitation is the difficulty of maintaining physiologically suitable and stable environmental conditions during prolonged measurements, particularly when using ultra-high-field preclinical MRI scanners that were originally developed for small-animal imaging.

In this work, we present a low cost, climate-controlled and MR-compatible growth chamber that includes an in-bore extension for preclinical MRI scanners. The system integrates growth and imaging conditions into a single setup, allowing continuous control of temperature, humidity, and illumination by the same system and removing the need to maintain separate commercial growth chambers alongside custom in-bore extensions. The implementation was optimized for the horizontal bore of a small animal scanner (Bruker PharmaScan 70/16) with 16 cm bore diameter and 72 mm free access but is applicable to other ultra-high-field preclinical MRI systems with comparable dimensions.

The performance of the climate chamber and the in-bore extension was characterized with respect to temperature, humidity, and illumination stability. In addition, the potential negative impact of the insert and its electronics on the MRI signal (B_0_ homogeneity, RF attenuation as well as potential RF artefacts) were verified.

Functional validation in form of sap flow measurements as well as anatomical validation was demonstrated in a naturally transpiring stem of *Passiflora quadrangularis*. Under controlled in-bore environmental conditions, changes in sap flow velocity were reliably detected using a pulsed field gradient spin-echo sequence. Specifically, increasing the light intensity in the extension resulted in a shift of the maximum flow velocity in individual vascular bundles from 0.21 mm/s and 0.39 mm/s to 1.37 mm/s and 1.17 mm/s, respectively. In addition, high-resolution anatomical imaging (1 mm slices with an in-plane resolution of 25 µm) of branching regions in *Dracaena braunii* was successfully performed without observable motion artifacts. The presented system provides a low-cost, open-source solution for conducting anatomical and functional MRI studies of intact plants using ultra-high field preclinical MRI scanners.

**Supplementary Information:**

The online version contains supplementary material available at 10.1186/s13007-026-01561-2.

## Introduction

Despite its considerable potential for non-invasive, three-dimensional anatomical and functional imaging, including the quantification of dynamic water transport in plant tissues, MRI remains an underutilized technique in botanical and biomimetic research [1–7]. However, its adoption is limited by the complexity of the technique and the high costs associated with both acquisition and maintenance of the necessary equipment.

Another challenge is the longer measurement time often necessary for high-resolution imaging or diffusion tensor imaging. This poses a significant challenge, as plants mounted inside the scanner are typically deprived of adequate water, nutrients, and light sources during the experiments. Water supply and humidity are especially critical, as dehydration-induced wilting can lead to movement artifacts, compromising data quality. When studying parameters beyond anatomical structure like sap flow or diffusion, precise control of environmental conditions becomes as important. Otherwise, fluctuations in light, temperature, and humidity can significantly affect measurement outcomes. To maintain stable and suitable conditions for repetitive and comparable measurements, customized MR-compatible climate-controlled chambers have been proposed before [5]. There are also distinct plant research dedicated high field MRI scanners that allow climate control but they are very rare [8, 9]. However, most studies are conducted at low field NMR spectrometers [3, 4, 10, 11] or at ultra-high field small animal MRI scanners, typically used for preclinical studies [2, 12–16].

Portable MR scanners represent another approach, enabling in situ imaging in natural outdoor environments or greenhouses with stable environmental conditions [1, 3, 7, 17–19]. Blystone et al. [20] provide a comprehensive review of portable MRI applications in plant research. Despite their advantages, portable MRI systems present certain limitations. Most rely on open-access permanent magnets [8, 21] which typically generate low magnetic field strengths, leading to a reduced signal-to-noise ratio (SNR). Consequently, both spatial and temporal resolutions are constrained, particularly in time-resolved studies.

An alternative approach is to integrate a climate-controlled chamber within the MR scanner. As no commercial solutions are currently available, we developed an MR compatible custom-built climate control chamber with extension for ultra-high field preclinical MR scanners. Our implementation was optimized for the horizontal bore of our Bruker PharmaScan 70/16 with 72 mm free access. It should, however, also work, or be at least easy to adapt, for any other ultra-high field preclinical MRI scanner with similar bore size.

Our design prioritizes cost efficiency and open-source accessibility, enabling other researchers to replicate the climate chamber and MRI extension, thus lowering the entry barriers to MR imaging of intact plants. The custom-built, climate-controlled growth chamber will constantly be connected to the extension, ensuring that plants remain in consistent environmental conditions before and during imaging, eliminating the need to transition from a commercial system to a custom in-bore extension with two independent control systems.

## Methods

### Hardware

A list of all components used is given in the supplements (Table S1) and is available on the GitHub entry of the project [22]. The mode of operation of the different regulatory systems is described in detail in the results part.

**Fig. 1:**
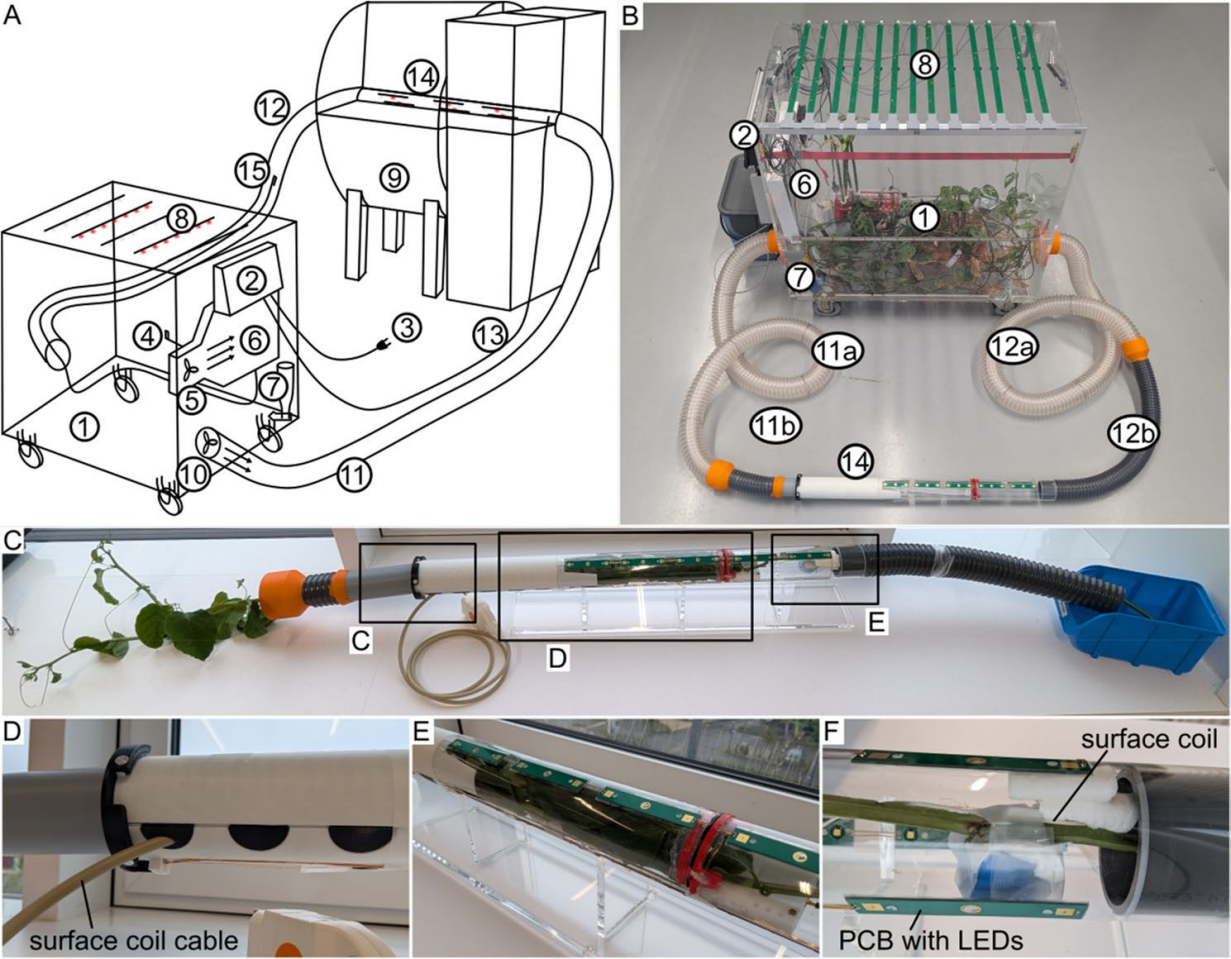
**A** schematic drawing of the main chamber (1) connected to the insert (14) installed in the MRI scanner (9). A high-pressure fan (10) generates a continuous air flow from the main chamber through the insert back into the main chamber, see arrows for flow direction. **B** Setup of the climate chamber connected to the extension outside of the MRI scanner, comprised of flexible tubes (11a, 12a), PVC adapter tubes (11b, 12b) and the insert (14). **C** A *P. quadrangularis* sample is mounted inside the insert; the PVC adapter tubes are installed. Care is taken that the cut end of the stem is submerged in water during mounting to avoid embolisms (blue container). **D** The extension cable of the surface coil amplifier is lead out of the extension through a 3d printed TPU sleeve. **E** The leaves are carefully folded so the surface is exposed to the LEDs. **F** Close-up of the stem centered in the extension with a surface coil mounted on top. Labeling: (1) main chamber (2), control panel (3), power cord (4), chamber sensor (5), circulation fan (6), heating unit (7), humidifier (8), LED strips chamber (9), MRI scanner (10), high pressure fan (11), connection tube (12), return tube (13), connection cord (14), MRI insert (15), insert sensor

Custom printed circuit boards (PCBs) for the control panel, the light-emitting diode (LED) strips of the main enclosure and of the insert were designed in OrCAD (Cadence Design Systems Inc., USA; Fig. 1A). Annotated circuit diagrams are attached in the supplements (supplementary material 1–4) and are available on the GitHub repository, together with the custom PCB layouts. The referenced components soldered onto the PCBs are listed in Table S1, all other components used are listed in Table S2.

Custom 3D-printed parts were designed in Autodesk Inventor and printed in polylactic acid (PLA) on a Raise 3D Pro3 printer (Raise 3D Technologies Inc., Netherlands). STP files of the climate chamber and the extension (Fig. 1A), containing all the custom printed parts in their designated positions, are available for download on the GitHub repository [22].

### Characterization

To characterize the performance of the climate chamber, five cycles changing the temperature and/ or humidity inside the chamber were conducted: [1] The temperature was set from 24 °C to 35 °C to test the performance of the heating unit and [2] the maximum humidity reached at 24 °C and 30 °C was tracked to test the performance of the humidity control system. Finally, the influence of the plant watering system on the stability of the humidity was tested. The watering system waters a total of 80 ml every 24 h in the current configuration. To quantify the difference in temperature and humidity between the chamber and the extension, measurements were taken inside of the chamber (Fig. 1A [1]) and in the return tube (Fig. 1A [12]). A custom Python script (Python 3.12, script provided on GitHub [22]) was used to read out the values measured by two Sensirion SHTC3 temperature & humidity sensors (Adafruit Industries, New York City, New York, United States) (Fig. 1A [4, 15]) during the cycles. The sensors have a measurement range of 0 to 100% relative humidity, -40 °C to 125 °C temperature range and a typical accuracy of ± 2% RH and ± 0.2 °C, respectively.

In addition, the maximum illumination intensity in both the main chamber and the extension was measured using a luxmeter (Extech instruments LT300 light meter) with measurement ranges of up to 0.04, 0.4, 4, 40, or 400 kilo Lux (klx) and an accuracy of ± (5% reading value + 0.5% measurement range). In order to adapt the illumination intensity in the extension, a passive diffusion layer consisting of one to three sheets of printing paper (80 g/m², ISO 100 brightness, 0.11 mm thickness) was introduced between the LEDs and the plant to modulate illumination intensity and ensure homogeneous light distribution within the extension.

### Validation

The extension was purpose built for a 7T Bruker PharmaScan 70/16 with horizontal bore. A quadrature volume coil with inner diameter of 72 mm was used for excitation together with a 2 cm surface loop coil (Bruker, Germany) for detection. To test the potential impact of the extension on image quality and on plant physiology, field maps and several imaging tests were completed.

The potential effect of the insert on field homogeneity was verified by acquiring field maps of a homogeneous water phantom with and without the insert. While imaging with the insert, we also tested the impact of the electronics of the chamber by comparing the field maps with electronics switched off and on.

Also, potential RF attenuation effects due to the insert material (acrylic) was tested at the location where plants were measured.

Furthermore, a 2D FLASH experiment was performed in the branching area of a *Dracaena braunii* specimen to test image quality and check if high-resolution, artefact free scans are possible. Scan parameters were: image acquisition time TA = 3 h, repetition time TR = 500 ms, echo time TE = 5.8 ms, 36 averages, 15 slices (1 mm thick) with a field of view FOV = 27 mm x 15 mm and an in-plane resolution of 25 μm.

Functional scans were also performed by measuring the sap flow velocity in a naturally transpiring stem section of *Passiflora quadrangularis* using a pulsed field gradient spin-echo sequence (PFG-SE) [3, 6, 23, 24]. Light was emitted to trigger photosynthetic activity and transpiration of water, increasing the sap flow in the xylem. Two scans with PFG-SE were executed during the night and with constant 24 °C temperature and 60% humidity. Scans were deliberately performed during the night to prevent a natural fluctuation in the sap flow velocities due to the circadian rhythm of the plants. The first scan was performed with the light turned off, the second scan with 5% LED intensity, corresponding to 9250 lx and 32,700 lx for the red and white channels respectively. Figure 1C-F show *P. quadrangularis* mounted in the insert ready to be installed in the MRI scanner.

Imaging scan parameters of the PFG-SE experiment were: TA = 5 min 10 s, TR = 1 s, TE = 60 ms, RARE factor of 10, FOV = 20 mm x 20 mm, 6 slices with 1 mm thickness and an in-plane image resolution of 200 μm. The flow encoding parameters were: gradient duration δ = 4 ms, diffusion time Δ = 50 ms, 1 A_0_ image followed by 30 b-values with maximal b-value = 4662.4 s/mm^2^, resulting in a maximal detectable displacement of 0.147 mm during diffusion time Δ and therefore maximal velocity of 2.94 mm/s along the diffusion encoding axis with 0.196 mm/s increments. For more theoretical information, refer to Callaghan et al. 1988 [25]. A 2D FLASH image was acquired as background reference.

## Results

### Hardware: enclosure and control panel

The main enclosure consists of a custom-made acrylic box (62 × 102 × 90 cm) with a lid and front door (Fig. 1A (1)). Both are fastened with generic bar hinges and can be opened for access. The lid is held in its open position using gas struts, the front door is kept shut using ball latches, a ratchet strap is used to prevent shearing when the box is moved around. Four casters with brakes are mounted under the enclosure so that it can be moved easily. 3D printed hooks on the backside of the enclosure are used to store the in-bore extension when not in use.

**Fig. 2:**
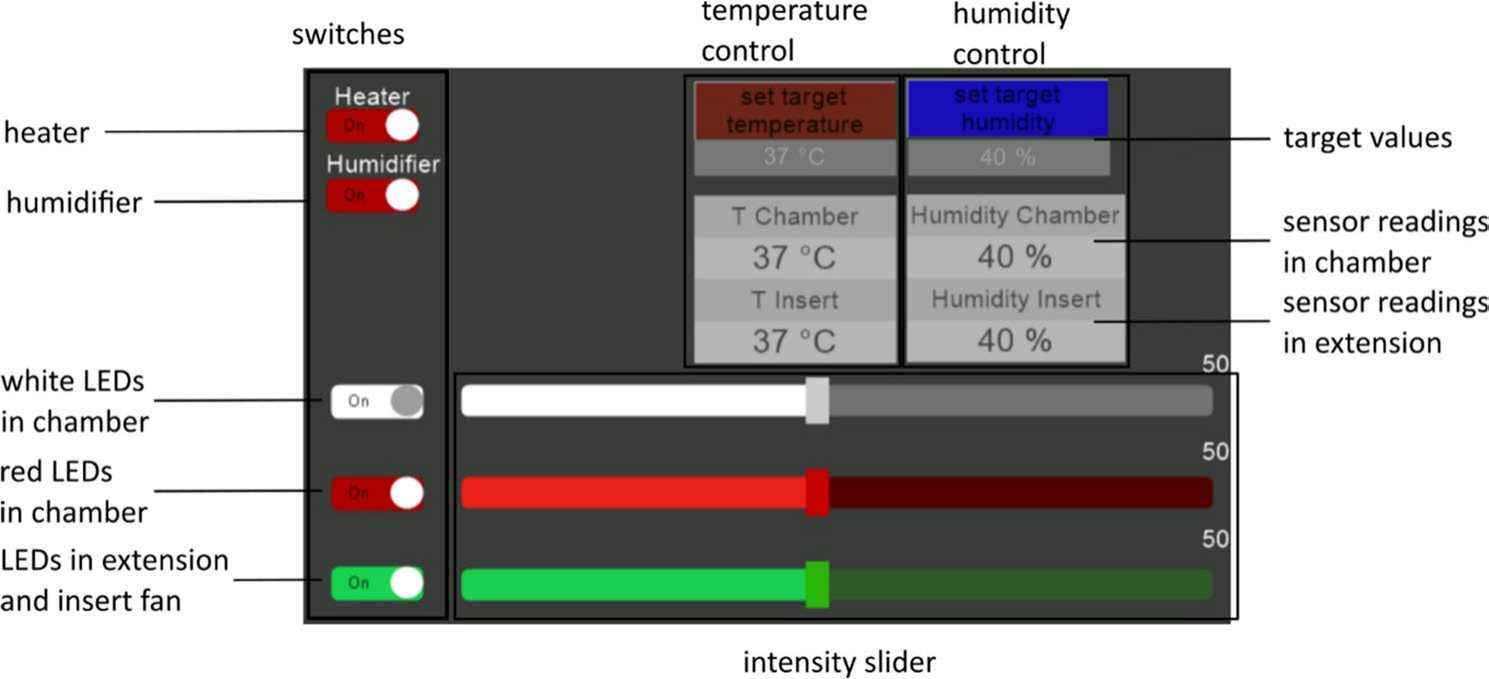
Layout of the control panel. The control panel consists of switches to control the different regulating systems (left side of the touch display). Temperature and humidity controls are located above the readings from the Adafruit Sensirion SHTC3 temperature & humidity sensors (top). The light intensity can be controlled with sliders (bottom). Lighting control is set to automatically switch off and on at 8:00 PM and 8:00 AM respectively

The control panel is mounted on one side of the enclosure and consists of an angled console housing with a resistive touch display for a better viewing angle (Fig. 1A (2) and Fig. 2). Software runs on a Teensy 4.1 microcontroller (SparkFun Electronics, Niwot, Colorado, United States).

Two AA batteries are connected to the VBAT and GND Terminals of the Teensy 4.1 for a constant power supply of the real time clock module, so that the time reference for the microcontroller keeps running and does not loose count of time when the climate chamber is plugged out.

The control system code was written using the Arduino integrated development environment (Arduino S.r.l., Monza, Italy, Arduino IDE, Version 2.3.8), and the display interface was designed using the proprietary Nextion Editor (Itead Studio, Shenzhen, China). All components and custom PCB layout details as well as the source files can be found in the Material and Methods section, the supplements, and are available on the GitHub repository.

### Hardware: temperature control

The temperature control system consists of an Sensirion SHTC3 temperature & humidity sensor (Adafruit Industries, New York City, New York, United States), a high flow, low-pressure circulation fan and an 800 W heating mat (Fig. 1A (4–6)). Preliminary experiments with a hot-air gun showed that heating power of about 800 W was necessary to heat the volume of the enclosure of approximately 0.5 m^3^ within a reasonable amount of time. Turned aluminum standoffs suspend the heating mat in an enclosure of high-pressure laminate (HPL) plates on either side and a 3D printed frame. The sensor is located on the 3D printed intake side in front of the circulation fan. The assembly is mounted on the inside of the enclosure (Fig. 1B (6)). In our case, target temperatures are between the ambient temperature of the laboratory and tropical temperatures, we do not need a cooling system at present.

### Hardware: humidity control

The humidity control assembly consists of an ultrasonic evaporator with a wick that soaks up water from a 3D printed siphon connected to a standard hard PET bottle (Fig. 1A (7)). As the hard PET bottle does not deform under the negative pressure, the water level in the siphon stays the same and the wick is always submerged in water.

### Hardware: lighting control

The lighting is comprised of two separate channels of white and red LEDs. The LEDs are passively cooled and are mounted on the outside of the lid so they do not introduce uncontrolled heat into the enclosure (Fig. 1A-B (8)). Each LED channels is supplied by separate power supplies, delivering 60 W with 12 V and 5 A. The LEDs are arranged on strips with 4 LEDs each, wired in series. Fuses (800 mA) are soldered in series onto the PCBs to prevent LED burnout. Eight strips are wired in parallel for the white channel and five strips are wired in parallel for the red channel. The brightness of both channels can be dimmed independently.

### Hardware: In-bore extension

The in-bore extension was designed to fit inside the rat volume coil (72 mm) of the Bruker PharmaScan 70/16 and consists of two different types of tubes that connect the extension with the main chamber (Fig. 1A-B). As an in- and outlet of the chamber, an ester-polyurethane suction and blower hose with a spring steel wire spiral is used (Fig. 1B (11a, 12a)). This tube is highly flexible and simplifies handling. However, since this tube is partly ferromagnetic, it is only used outside the fringe field of the magnet. To connect the flexible tube with the insert, a soft PVC tube with a hard PVC spiral is used (Fig. 1B (11b, 12b)). A secondary temperature & humidity sensor (Adafruit Sensirion SHTC3) is individually placed inside the return tube measuring the conditions in the extension (Fig. 1A (15)).

A high pressure, high flow fan was used instead of an independent heating and humidity control system, to circulate the air from the main chamber through the extension and back into the main chamber (Fig. 1A (10)). The benefit of this construction type is the reliable stability of the climate condition in the extension when air is pumped from the large air volume of the main chamber into the much smaller extension unit.

The lighting of the insert is controlled independently from the main chamber by the touch screen control panel (Fig. 2). However, unlike for the main chamber, the white and red channels are combined into one circuit, to reduce the amount of wiring (and power supplies) needed (Fig. 1E-F). A 90 W, 42 V, 2.15 A power supply was used to provide power to 3 parallel strips of 15 LEDs, each wired in series. On every strip, there are two white and one red LED. The acrylic tube was notched with a CNC milling machine and annealed afterwards to fit the custom PCBs for the LEDs on the outside of the tube. This keeps the outer diameter of the insert unchanged, so that it easily fits into the bore of the MR scanner. In Addition, the PCBs do not protrude far into the interior of the acrylic tube, which would limit the space for samples (Fig. 1E-F).

The top part of the insert can be partly removed for convenient access. Plugs are used to connect the circuitry and hold the removable piece in place. This simplifies the placement of the sample and the surface coil in the insert (Fig. 1C-F). The surface coil is mostly used for smaller samples or plant parts of interest, larger samples that fill out the bore (depending on the filling factor) are imaged using the volume coil. Cutouts at one end of the insert allow the insertion of the surface coil cable (Fig. 1D). Furthermore, these cutouts may be used to insert the stem of bigger plants, should the root ball be too big to fit inside the connection tubes. A connection cord (Fig. 1A (13)) to the control panel can be plugged into the insert, once everything is positioned properly.

### Characterization: temperature and humidity regulation

The parameters and results of the five tests performed to characterize the performance of the temperature and humidity regulation systems are summarized in Table 1.

**Table 1:** Testing scenarios performed to characterize the performance of the temperature and humidity regulation systems with the set values, the stable conditions reached in each test (mean) and the time it took to reach stable conditions. Accuracy of the Adafruit Sensirion SHTC3 Temperature & Humidity Sensor was specified by the manufacturer as ± 2% relative humidity and ± 0.2 °C

test scenario	set temperature	set humidity	values chamber	values extension	time to stable conditions
1) max humidity at 24 °C	24 °C	100%	24 °C 80%	-	87:30 min
2) max humidity at 30 °C	30 °C	100%	30 °C 67%	-	46 min
3) effect of watering on humidity	24 °C	60%	before: 24 °C 66% after: 24 °C 66%	-	-
4) chamber vs. extension	24 °C	60%	24 °C 60%	23 °C 54%	24:30 min
5) max heating capacity	35 °C	60%	35 °C 51%	-	4:30 min

**Fig. 3:**
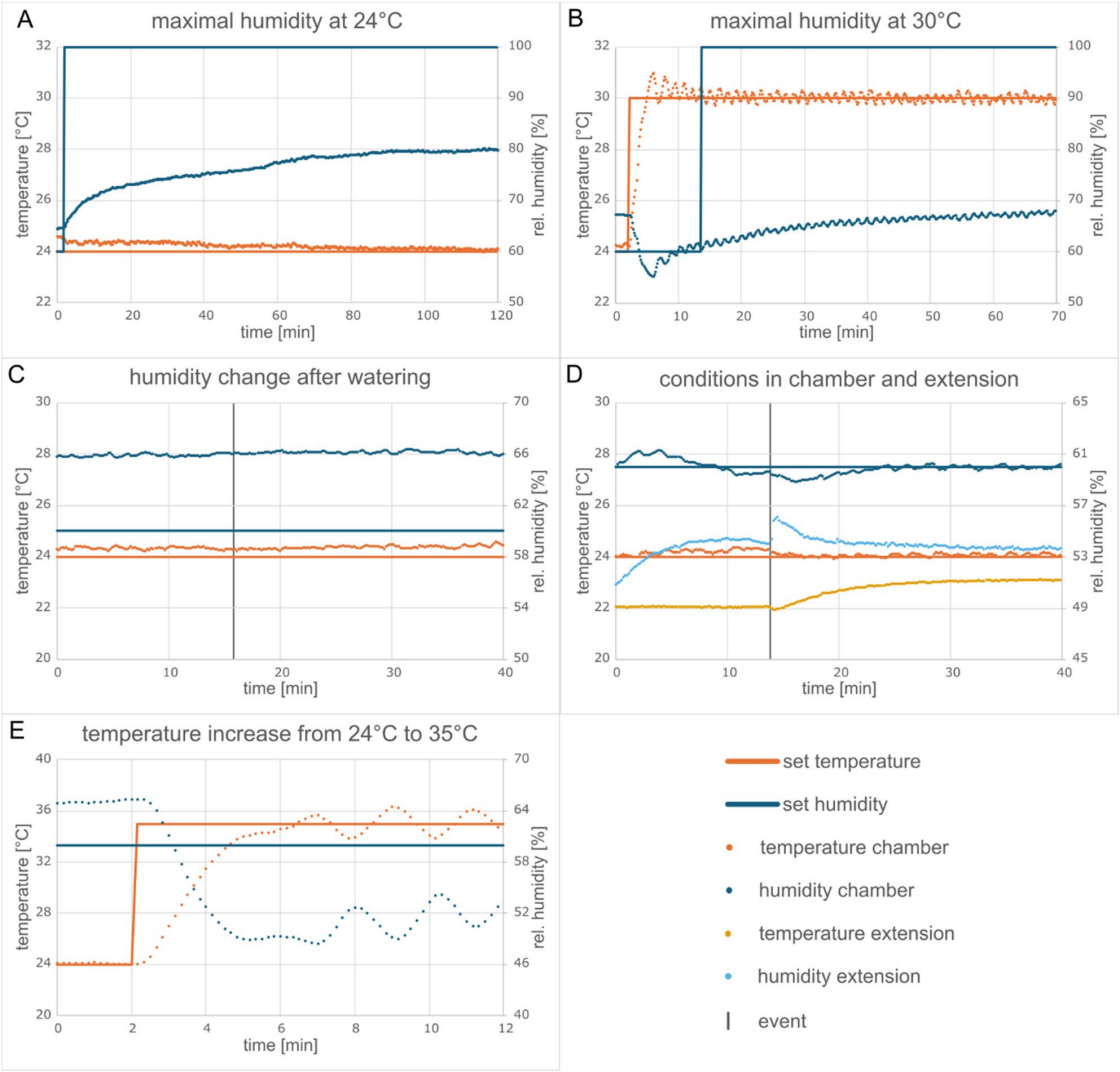
Course of temperature (temp) and relative humidity (rh) of the five testing scenarios. **A** The set rh was changed from 60% to 100% at constant temp of 24 °C. A maximum rh of 80% was reached after 87:30 min. **B** The set temp was changed from 24 °C to 30 °C and the set rh from 60% to 100%. With rising temp, rh dropped to 55%. After chamber rh went back up to 60%, the set rh was changed to 100%. Rh of 67% was reached after 46 min. **C** Introduction of extra water (in form of wet soil) into the climate chamber trough the automated watering system (grey line) does not considerably change temp and rh. **D** Temp and rh in the main chamber and the extension before and after turning on the circulation fan (grey line). Conditions were stable at 24 °C and 60% for the chamber and 23 °C and 54% for the extension. **E** Ramp up test after changing the set temperature from 24 °C to 35 °C. Sinusoidal fluctuations occur when 35 °C is reached. The relative humidity reacts to the temperature fluctuations and decreases as the temperature increases and vice versa

To characterize the temperature and humidity regulation, the Adafruit sensors was read out in five scenarios (Fig. 3A-E).

In the first two experiments, the maximum achievable humidity at 24 °C and 30 °C were determined by changing the set humidity for both temperatures from 60% to 100%, measuring until the humidity level stabilized (Fig. 3A and B). This revealed that a humidity of 80% was reached at 24 °C after changing the set temperature to 100%, which took 87:30 min (Fig. 3A).

In preparation of the second experiment, determining the maximum humidity at 30 °C, the temperature was changed from 24 °C to 30 °C (Fig. 3B). The target 30 °C was reached rapidly (2:50 min), thereby inducing a drop in humidity to 55%. Therefore, we waited for the measured humidity to reach 60%, before setting the humidity to 100% in the second experiment. A stable humidity of approx. 67% was obtained 46 min after changing the humidity set point to 100%.

In the third experiment, we tested the stability of the chamber humidity following the introduction of additional water in the soil through the automated watering system (Fig. 3C). The temperature and humidity changed only slightly after watering, with mean values remaining stable at 24 °C and 66%, respectively.

For the fourth experiment, the temperature- and humidity-drop from the main chamber to the extension was quantified (Fig. 3D). The humidity in the extension rises in the first 14 min from 51% to 54% as the humid air from the chamber slowly enters the newly connected tubes that are still filled with laboratory air. The vertical line at 14 min in Fig. 3D indicates the point where the circulation fan was turned on. In the following, a rapid humidity increase to 56% is detected in the extension, before stabilizing at 54%. In the main chamber, a slight drop in the humidity to 59% can be detected around the vertical line at 14 min. Subsequently, the main chamber humidity stabilizes at 60%. The temperature in the extension slowly increases to a stable 23 °C while it stays at a constant 24 °C in the main chamber.

Finally, the maximum heating capacity was tested by setting a target temperature of 35 °C (Fig. 3E). A temperature of 35 °C was reached 4:30 min after changing the set temperature from 24 °C to 35 °C. After that, the temperature fluctuates by approx. 1 °C around 35 °C in a sinusoidal pattern. Parallel to the rising temperature, the humidity dropped from 65% to 49%. It then fluctuated around 52% (+/-2%), alternating with the temperature fluctuation.

### Characterization: Lighting in climate chamber and extension

To characterize the lighting in the climate chamber and the extension, the illuminance is measured at the bottom, at the top and at medium height for different power levels using a lux meter (see Table 2). Inside the extension, measurements were performed in the direct vicinity of the LEDs, where the plant’s leaves would be expected to be located. The maximal illuminance measured in the climate chamber was 3752 lx, 8640 lx and 21,070 lx at the bottom, at medium height and at the top, respectively, with the LED intensity set to 50% for both channels. Power supply levels above 50% were avoided because they result in the LEDs becoming too hot as they are only passively cooled. The extension reached even higher values with up to 122,900 lx and 213,000 lx under the red and white LEDs, respectively. Due to the proximity of the LEDS to plant leaves, we tested lux values with 5% power resulting in 19,250 lx and 32,700 lx for red and white LEDs, respectively. In order to diffuse the light and reduce the illumination intensity further in order to reach different climatic conditions, filters were applied. The use of several standard sheets of printing paper have been proven to be sufficiently effective whereby one, two and three layers were tested, corresponding to 0.11 mm, 0.22 mm and 0.33 mm filter thickness (Table 2). Three layers with 5% power reduced illumination intensity from 19,250 lx to 1025 lx and from 32,700 lx to 1523 lx for the red and white LEDs respectively.

**Table 2:** Peak illuminance measurements in the main chamber and extension at different power settings (indicated in lux). The measurements in the main chamber were taken in the center of the chamber at the bottom, vertically centered and on top, for the extension directly under the white and red LEDs. Standard printing paper with up to 3 layers was used to diffuse and reduce illumination intensity in the extension

LED power level measurement setup			5%	10%	20%	30%	40%	50%
climate chamber	bottom	Red	142	218	401	548	629	857
		White	231	538	1218	1764	2341	2906
		Combined	372	794	1663	2390	3098	3752
	centered	Red	405	557	1008	1120	1660	2268
		White	511	1247	2590	3700	4990	6119
		Combined	928	1841	3655	5230	6920	8640
	top	Red	683	833	1550	2180	3260	4850
		White	1006	2499	4860	7370	10,090	15,830
		Combined	1637	3370	6510	9750	13,280	21,070
extension	no filter	Red	19,250	31,600	55,900	81,000	102,400	122,900
		White	32,700	55,100	98,400	139,100	176,100	213,000
	one layer	Red	4340	7320	12,980	18,660	23,450	28,100
		White	7910	15,000	26,280	36,020	46,900	56,400
	two layers	Red	1630	2820	5210	7550	10,080	12,100
		White	3240	5530	10,230	13,850	18,270	21,800
	three layers	Red	1025	1597	2440	3450	3840	5740
		White	1523	2402	3980	5810	7180	9520

### Image quality validation

All B_0_ field maps were very similar: Homogeneity was neither altered by the insert itself nor by the switch of the LEDs (Fig. S1). We notice here that the field of view is logically located next to the PCBs and not directly underneath. Similarly, the RF attenuation measured with the insert installed and the LEDs turned on was < 5%.

**Fig. 4:**
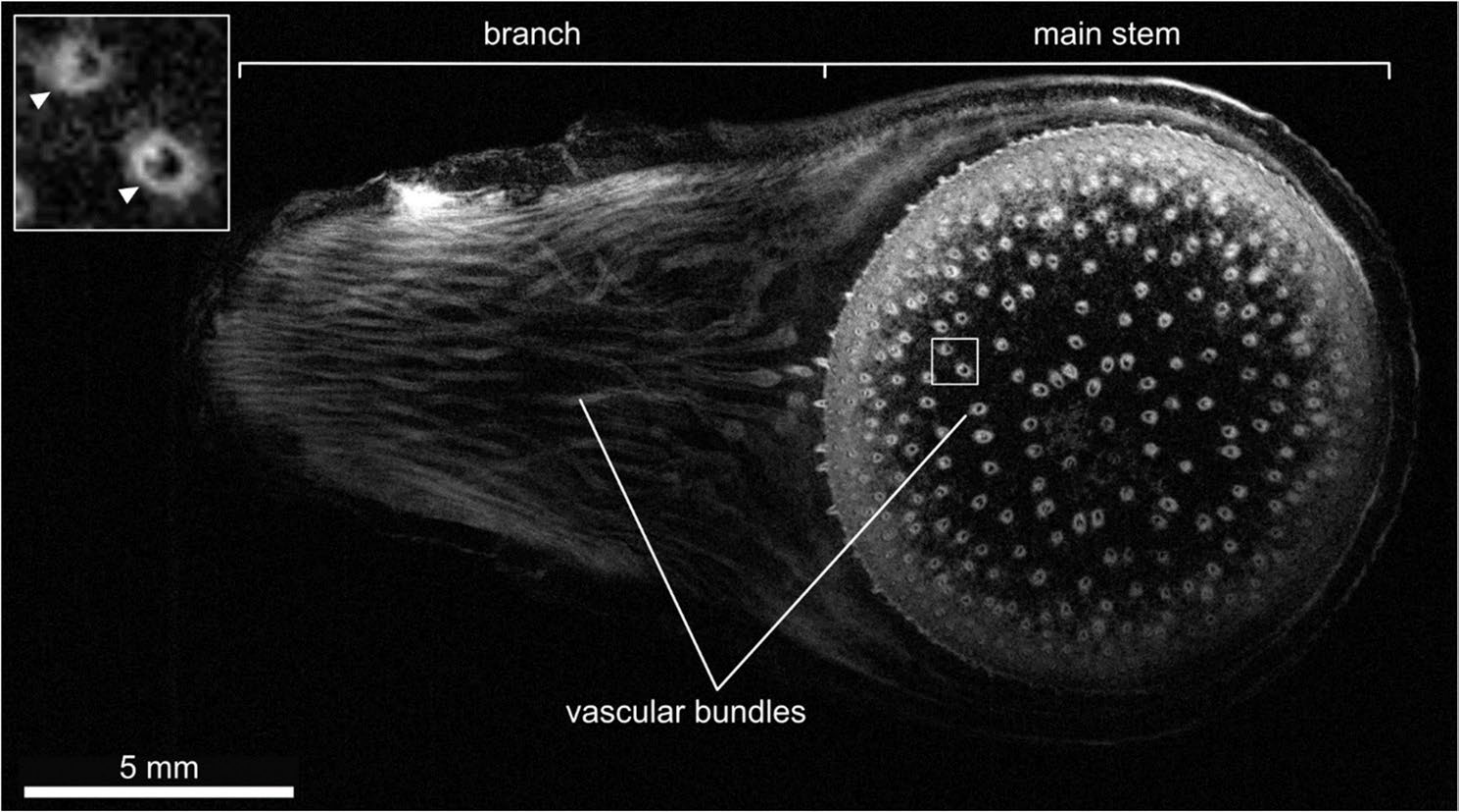
Anatomical FLASH scan within the branching region of a *D. braunii* mounted inside of the extension. The highlighted square on the top left side shows a closeup of the two vascular bundles (position highlighted). The cross-sectional slice is located in the middle of the attachment area of a branch that attaches to the main stem. Individual vascular bundles in the main stem and the branch are visible (arrows in highlighted square). Slice thickness 1 mm, 25 × 25 μm in-plane resolution. Climatization parameters during the scan were set to 24 °C, 60% relative humidity and 5% illumination power

Additionally, in order to test if sharp high-resolution, artefacts free scans are possible while using the extension, we performed an anatomical scan of *D. braunii* (compare Fig. 4 with [2, 14]). Despite the long acquisition time (3 h), neither motion-related smearing artefacts nor any type of electronics induced RF artifacts were observed. Figure 4 shows slice 9 of 15, located at the attachment zone of a branch (left) to the main stem (right). The lower signal intensity toward the bottom of the image is in this case attributable to the surface coil. With a slice thickness of 1 mm and an in-plane resolution of 25 μm, individual vascular bundles were clearly resolved in both the branch and main stem.

**Fig. 5:**
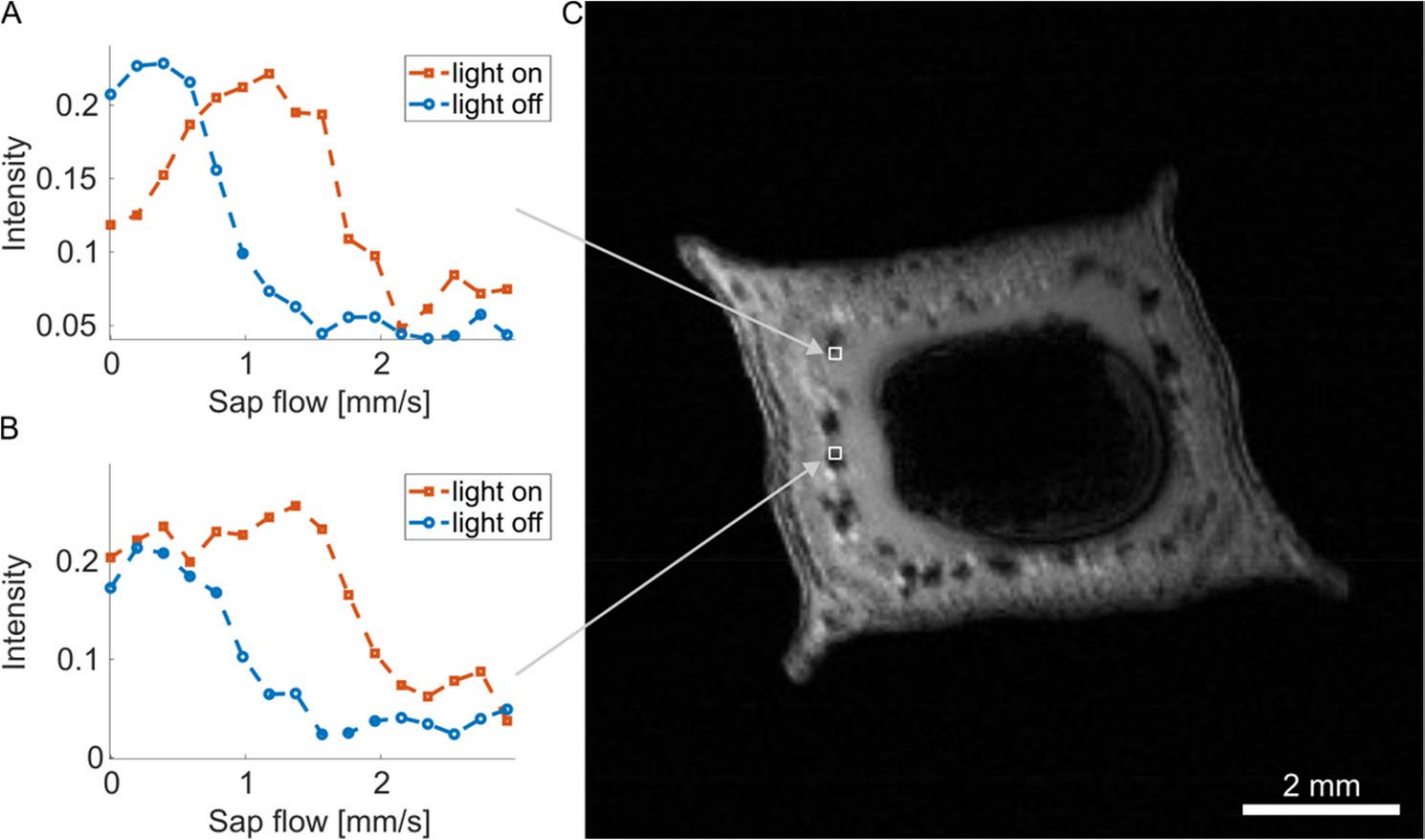
Functional imaging of sap flow velocity in a *P. quadrangularis* stem using a pulsed field gradient spin-echo sequence (PFG-SE). **A** and **B** show the sap flow velocity profile in two representative voxels in the xylem with the light in the climate chamber extension turned on (orange) or off (blue). The mean velocity, indicated by the peak in the profile, shifted from 0.21 mm/s to 1.37 mm/s (**A**) and from 0.39 mm/s to 1.17 mm/s (**B**). Voxel position is marked in the anatomical FLASH image (**C**)

To test the functionality of the extension on plant physiology during scanning, we conducted sap flow measurements using a pulsed field gradient spin-echo sequence in a naturally transpiring stem section of *P. quadrangularis*. Figure 5A-B show the measured sap flow velocity profiles in two different voxels with the lights turned on and off. The location of the voxels is marked in the FLASH image in Fig. 5C. As would be expected, a clear change in sap flow velocity can be detected with a shift in the mean velocity (indicated by the maximum intensity) from 0.21 mm/s to 1.37 mm/s in Fig. 5A and from 0.39 mm/s to 1.17 mm/s in Fig. 5B. To assure that the increased flow velocity measured was caused by the lights in the extension and not the surrounding daylight or the circadian rhythm of the plants, “lights on” and “lights off” experiments were both performed during the night with no surrounding light sources.

In the anatomical scan of *P. quadrangularis*, the relatively large slice thickness caused the signal in each voxel to be averaged over 2 mm resulting in partial volume effects, meaning a blurred image with reduced sharpness.

## Discussion

### Advantages and limitations

The main advantages of the MR compatible climate chamber design are the low-cost and the easy availability of all components and material used while ensuring functionality and practicability of both climate chamber and in-bore extension. The most expensive item is the acrylic enclosure with acrylic glass covering all sides. This design was applied to enable unobstructed observation and inspection of the plant development and condition inside the chamber without having to open the chamber which would lead to climate fluctuations. In addition, growth can be videotaped and photographed from outside the chamber through the acrylic glass preventing equipment corrosion at high relative humilities. A viable alternative would be to screw acrylic panels onto a frame made of aluminum profiles, which would cost equally or less than a glued enclosure, depending on material prices. This would additionally increase the torsional resistance of the enclosure, reducing the risk or crack formation under heavy load on uneven surfaces.

The overall usability of the climate chamber is intuitive and sufficient for the applications presented in this study. Sample preparation can nevertheless be challenging, as preclinical scanners are not purpose-built for imaging living plants under controlled environmental conditions, in contrast to dedicated plant-MRI systems reported elsewhere [3]. Conventional clinical and experimental MR scanners are not designed to accommodate extensive plant preparation, climatization, and illumination during imaging. As a result, plants may experience mechanical stress, such as damage to stems, surfaces, or leaves, as well as prolonged preparation times and insufficient resource supply. The developed extension enables sample preparation and climatization before insertion of the plant into the bore. In particular, the opening in the insert facilitates sample handling by providing easy access for positioning the plant. However, the extension is designed for a horizontal bore geometry and the potential influence of gravitropism must be considered. Even if those effects are suspected to be negligible during the typical acquisition time of an MRI scan and especially for anatomic imaging, physiological processes such as sap flow dynamics might be influenced [26]. *P. quadrangularis* used for sap flow analysis in this study is a climbing plant and stem parts can grow horizontally. Thus, we expect no sufficient influence of the horizontal placement on sap flow dynamics in climbing species.

#### Lighting

LEDs used for plant illumination need to exhibit wavelengths within the photosynthetically active spectrum, especially the absorption spectra of chlorophyll a and b. The lower absorption spectra of chlorophyll a and b at 430 nm and 453 nm, respectively, are covered by the emission spectrum of the white LEDs used. To cover the higher absorption spectra of 662 nm and 642 nm, red LEDs with an emission peak at 655 nm were added. In addition, typical values for light intensities are ~ 2000 lx in the undergrowth of tropical rainforest and in temperate zones [27], 2000–5000 lx on an overcast winter day, 10,000–20,000 lx on an overcast summer day and > 100,000 lx on a clear summer day.

To ensure full coverage of these values within the climate chamber, both the positioning in the chamber as well as the power settings of the LEDs must be considered: undergrowth of tropical rainforest (2000 lx): bottom of chamber 20–30% power; overcast winter day (2000–5000 lx): middle of chamber, 10–30% power; overcast summer day (10000–20000 lx): top of the chamber, 40–50% power. To simulate lighting conditions on a clear summer day (> 100000 lx), the LEDs would need active cooling to handle power levels of over 50%. Depending on where the chamber is set up, ambient light from windows or ceiling lights can change the illuminance inside the enclosure.

A proper illumination of plants inside the bore is more complex. Other studies have described plant illumination using four LED strips mounted within a semicircular tube positioned outside the bore, providing light from below [28]. The main limitation of this configuration is the space it would occupy within the bore. As sample preparation is already challenging, often requiring simultaneous handling and control of multiple branches, leaves and other laterals facing in all directions, further reducing the limited available space complicates plant positioning and increases the risk of tissue damage. Our approach consists of individual LEDs soldered onto thin PCBs that are recessed into the acrylic tube of the insert from the outside. Holes were drilled for the LEDs such that they protruded only minimally into the interior of the acrylic tube, enabling illumination of the plants from all sides during installation in the scanner. Connectors integrated into the detachable lid allow removal of the upper half of the acrylic insert tube which also includes the LEDs. This drastically simplifies the positioning of the plant and the volume coil inside the extension. The use of individual LEDs has some limitations. Leaves in close proximity to the LEDs may be damaged by heat if appropriate operating conditions are not maintained. In addition, because light is emitted from discrete point sources, leaves located near the LEDs are more susceptible to photoinhibition. The threshold at which photoinhibition occurs depends strongly on the plant species and on the light intensity to which the plant is adapted. Experiments show that sun-adapted species like beech only exhibit improved photosynthetic performance at over 2000 lx [29], while shade-adapted understory species such as *Aeschynanthus longicaulis* experience significant photoinhibition at 650 µmol m⁻² s⁻¹ PPFD [30]. A direct conversion of PPFD to illuminance is spectrum-dependent; for typical white LED light, 650 µmol m⁻² s⁻¹ corresponds to approximately 42,000–48,000 lx. This value is already reached at 10% power in the extension, when not using paper to diffuse the light. Using printing paper as a filter helps to diffuse the light and reduce the peak intensity. In addition, it is a cheap, readily available filter option and does not take up much space. It is also easy to stack multiple sheets of paper, should the intensity be too high for the planned experiment. Depending on the desired conditions during scanning we recommend the following set of parameters: undergrowth of tropical rainforest (2000 lx): 5–10% power with 3 paper sheets, 30 °C, 70% humidity or higher; overcast summer day in temperate region (10000–20000 lx): 10–20% power with one paper sheet, 24 °C, 60% humidity; clear summer day in the mediterranean (> 100000 lx): >20% power without paper, 30 °C, 60% humidity.

#### Temperature and humidity regulation

The heating plate works adequately for the intended temperature range of up to 35 °C. However, as the plate becomes hot, we recommend to be sensible with temperature input as setting higher temperatures could cause the enclosure to melt. Once the set temperature has been reached, overshoots and sinusoidal fluctuations can be observed. These shifts can be easily explained through the inertia of the system and the frequency of the temperature regulation, as the heating plate is either switched on or off, depending on weather the target temperature is reached or not. Intentionally, the temperature cannot be lowered below room temperature since this functionality would increase the price of the chamber. The setup in this work did not require temperatures lower than typical lab temperatures. However, if a fine-tuned heating and humidification system is required, commercial laboratory humidity/temperature generators exist with varying flow rate, humidity/temperature range, and application focus. The aim of this study was the development of a low-cost approach. If precise and independent control of both humidity (5–95%) and temperature (5–75 °C) is required, specialized and modular humidity generators are required which can increase cost drastically by 20.000 € and more, i.e. MHG100 of ProUmid the F-series of Cellkraft (without cooling capabilities) or the CMS 400 of Heinz Walz GmbH used by Rokitta et al. [31]. In contrast, the temperature and humidity control system costs of the device developed in this study lies under 200 €, including sensors, hardware and the 3d printed parts. With a limited budget, this system is sufficient for controlling conditions ranging from temperate to tropical climates [32]. The focus of this work was on low-cost alternatives for a functional climatization device compatible with MRI. Hence, we deliberately chose a low-cost ultrasonic evaporator as basis for the humidity control system which has two limitations: (1) The maximum achievable humidity imposes a primary limitation. Because the saturation concentration of air increases exponentially with temperature, higher temperatures require more water vapor to reach the same relative humidity. This is constrained by the capacity of the ultrasonic evaporator. While adding a second ultrasonic evaporator could overcome this limitation, the current setup provides a sufficient humidity range for our purposes. As mentioned above, commercial lab systems exist, which allow more precise humidity regulation at much higher costs. (2) Humidity cannot be lowered below the ambient room level outside the chamber. Two approaches can reduce chamber humidity. A low-cost option is to use replaceable desiccant media, such as silica gel or dehumidifying salts, through which air is passed when dehumidification is needed. The connection to the chamber can be disengaged when not in use. Alternatively, a condensation-based dehumidifier could be integrated. While a combined humidifier–dehumidifier unit would allow automated control of both humidity increase and decrease, such systems are typically expensive. Using a standalone dehumidifier is simpler, but would require manual adjustment of both the humidification and dehumidification systems.

#### Plant scans

The climate-controlled chamber and in-bore extension maintained stable environmental conditions, allowing plants to remain physiologically stable during scanning. This enables high-resolution anatomical imaging without visible motion artefacts, even for scan durations of up to 3 h. Altering the lighting inside the extension resulted in measurable changes in sap flow velocity, demonstrating that functional scans can be conducted under controlled conditions. As the other environmental parameters were held constant, this effect can be attributed to the applied illumination. Furthermore, both “lights on” and “lights off” scans were performed at night, ensuring that the observed changes in sap flow velocity resulted from photosynthetic activity rather than circadian rhythm.

### Design guidelines for in-bore hardware

All in bore materials were chosen to be non-magnetic and, if possible, non-conductive to reduce the occurrence of eddy currents. Acrylic was used for the insert as the same magnetic susceptibility is similar to water and thus does not induce artefacts [33]. An additional focus was on keeping the insert as flush against the bore walling as possible. To achieve this, a comparatively large effort was put in the machining of the acrylic insert tube and PCBs were ordered at thicknesses of 0.8 mm.

Copper and tin alloys were used for electrical connections. The LEDs were tested in-bore using a laboratory power supply while scanning phantoms with the sequences later used for imaging to ensure image quality was unaffected. Low-pass filters were put on the LED power supply outputs to reduce any noise or frequency peaks being carried into the bore through the connection cord. Pulse-width modulation controllers for fans were set to 100% or 0%, as it has been found that intermediate duty cycles cause banding artifacts.

## Conclusion

We present a low-cost, open-source climate chamber with an MR compatible in-bore extension designed for use with a horizontal small-animal magnetic resonance scanner. The system enables independent control of lighting in both the main chamber and the extension, as well as regulated humidity and temperature. Although it lacks the refinement of a dedicated plant-MRI system, it provides a cost-effective solution for controlling the key environmental parameters required for imaging living plants. By maintaining plant vitality during scanning, the chamber extension supports high-quality anatomical imaging. Moreover, the ability to adjust individual environmental variables allows functional imaging outputs, such as sap-flow-velocity maps, to be directly related to specific experimental conditions.

## Supplementary Information

Supplementary Material 1.

## Data Availability

No datasets were generated or analysed during the current study.

## Abbreviations

MRI: Magnetic resonance imaging
PFG-SE: Pulsed field gradient spin-echo sequence
TA: Acquisition time
TE: Echo time
TR: Repetition time
RARE: Rapid Acquisition with Relaxation Enhancement
FLASH: Fast Low-Angle Shot
PLA: Polylactic acid
PET: Polyethylene terephthalate
PVC: Polyvinyl chloride
PCB: Printed circuit board
LED: Light-emitting diode
SNR: Signal to noise ratio
