# Supplementary material for: In-bore climate control chamber for magnetic resonance imaging of living plants
Source: Plant Methods. 2026 Jul 7;22:60. doi: 10.1186/s13007-026-01561-2 (PMC13339381; doi:10.1186/s13007-026-01561-2)
Supplement: Supplementary file 1 — Supplementary Material 1. [file 13007_2026_1561_MOESM1_ESM.docx]

Supplementary for: In-bore climate control chamber for magnetic resonance imaging of living plants.

Supplementary tables

Table S 1: List of parts soldered onto PCB of control panel with quantity, reference on circuit and part designation. Annotated circuit diagram of the PCB in PDF as Supplementary material 1: PCB-layout-control-system. An additional expansion board was used for LED dimming, connected through X6 and X18. Annotated circuit diagram of the PCB in PDF as Supplementary material 2: PCB-layout-adapter-dimming.

| Item | Quantity | Reference | Part |
| --- | --- | --- | --- |
| 1 | 8 | C1, C2, C27, C28, C31, C32, C33, C34 | 470 nF |
| 2 | 9 | C3, C6, C8, C14, C29, C35, C41, C43, C45 | 1 uF |
| 3 | 9 | C4, C7, C9, C15, C30, C36, C42, C44, C46 | 0.1 uF |
| 4 | 1 | C5 | optional |
| 5 | 3 | C19, C21, C22 | 15 nF |
| 6 | 1 | C20 | 0.15 uF |
| 7 | 4 | X1, NTC, DIM_W, DIM_R | WAGO237-102 |
| 8 | 2 | D2, D3 | 1N4007 |
| 9 | 5 | Fan1, Fan2, Fan3, X17, Fog | 2-pin post |
| 10 | 1 | F1 | 1A/F |
| 11 | 8 | US1, H1B, H1A, US2, H2B, H2A, V~, N | flat plug 6.2mm |
| 12 | 4 | IC1, IC4, IC5, IC6 | TPSI3050DWZR |
| 13 | 1 | IC2 | AMC23C10 |
| 14 | 1 | J1A | SMD socket |
| 15 | 6 | MFET1, MFET2, MFET3, MFET4, MFET5, MFET6 | IPDQ60R020CFD7 |
| 16 | 4 | Q1, Q2, Q3, Q4 | FDPF320N06L |
| 17 | 1 | R1 | 10 Ω |
| 18 | 4 | R2, R9, R30, R31 | 11 kΩ |
| 19 | 5 | R3, R4, R5, R6, R7 | 510 kΩ |
| 20 | 9 | R21, R23, R25, R37, R39, R40, R42, R43, R45 | 10 kΩ |
| 21 | 1 | R22 | 220 Ω |
| 22 | 1 | R24 | 1 MΩ |
| 23 | 1 | R26 | 3.6 kΩ |
| 24 | 1 | R27 | 6.2 kΩ |
| 25 | 1 | R28 | 3 kΩ |
| 26 | 1 | R29 | 5.6 kΩ |
| 27 | 4 | R33, R34, R35, R36 | 10 kΩ |
| 28 | 2 | R38, R44 | 3.3 kΩ |
| 29 | 1 | R41 | 100 kΩ |
| 30 | 1 | U1 | AD8495C |
| 31 | 1 | U4 | OPA314AIDCK |
| 32 | 1 | U6 | Teensy 4.1 |
| 33 | 1 | U7 | 74HC4049 |
| 34 | 1 | U10 | TMA 2405S |
| 35 | 1 | U11 | PSK-40D-24 |
| 36 | 1 | U15 | TMA 2412D |
| 37 | 4 | U16, U17, U18, U19 | MCP14A1202-E/MS |
| 38 | 1 | U23 | OPA210IDT |
| 39 | 1 | U24 | OPA4244EA |
| 40 | 2 | X6, X18 | 4-pin Post |
| 41 | 1 | X7 | 3-pin Post |
| 42 | 4 | X8, X9, X10, X11 | 2-pin solder bridge |
| 43 | 2 | X15, X16 | 5-pin Post |
| 44 | 4 | DIM_W_Ext, DIM_W, DIM_R_Ext, DIM_R | DG350 |
| 45 | 4 | R1,R4,R6,R8 | 1,8k |
| 46 | 4 | R2,R3,R5,R7 | 3,6k |
| 47 | 4 | U1,U2,U3,U4 | OPA192-Q |
| 48 | 2 | X6,X18 | 4-pin Post |
| 49 | 1 | X20 | directFog |
| 50 | 2 | X21,X22 | 1-pin Post |

Table S 2: List of all parts used in the main climate chamber and the extension, sorted by the corresponding control system with designation, the feature why we chose the specific component, a link to the product or the file and the price.

| Enclosure | | | |
| --- | --- | --- | --- |
| Designation | feature | Source (link to product or file) | Price |
| Acrylic box | Enclosed box, viewable from all sides | Custom ordered | 1996.23€ |
| Bar hinge | Hinge | <https://www.hornbach.de/p/stangenscharnier-20x900-mm-vermessingt-1-stueck/5795409/> | 4.25€ |
| Ball latch | Hold front door in closed position | <https://www.hafele.com.de/de/product/doppel-kugelschnaepper-zum-schrauben/24420017/?MasterSKU=P-00871842> | 9.30€ |
| Gas struts | Hold lid in open position | <https://www.gasfedershop.de/produkt/6mm_kolbenstange_200mm_hub/> | 91.26€ |
| M5 hinge eye with angle fitting | Mount gas struts | <https://www.gasfedershop.de/produkt/m5-gelenkauge-16mm-mit-winkelbeschlag/> | 15.98€ |
| M5 hinge eye with side fitting | Mount gas struts | <https://www.gasfedershop.de/produkt/m5-gelenkauge-staerke-6mm-loch-6-1mm-mit-seitenbeschlag/> | 12.58€ |
| Wheels | To move the box | generic smooth-running swivel castor |  |
| Hooks | to store in-bore extension when not in use | 3D-printed (STEP file) |  |
| Control Panel | | | |
| Designation | feature | Source (link to product or file) | Price |
| Console housing | Angled for better viewing | <https://www.bopla.de/en/enclosure-technology/alu-topline/enclosure-7/enclosure-profiles-horizontally-divided-7/atph-1865-0300> | 88.54€ |
| Display | Usability, number of elements displayed, resistive touch due to water | <https://nextion.tech/datasheets/nx8048p070-011r/> | 86.00€ |
| Hardware for control panel | Control of electronics | wiring diagram (Supplementary material 1 and 2), list with parts (Table S 1) |  |
| Temperature control | | | |
| Designation | feature | Source (link to product or file) | Price |
| Circulation fan | High flow, low pressure | <https://www.conrad.de/de/p/sunon-eec0252b2-000u-a99-axialluefter-24-v-dc-157-97-m-h-l-x-b-x-h-25-x-120-x-120-mm-2992666.html> | 10.92€ |
| Air sensor | Integrated temperature and humidity with appropriate sensitivities | <https://www.adafruit.com/product/4636> | 6.95€ |
| Heating mat | 800W for sufficient heating power for a volume of 0.5 m^3^ to reach a Δt of 20° for half an hour | <https://www.roboter-bausatz.de/p/silikon-heizmatte-heizbett-220v-750w-310-x-310-mm-fuer-3d-drucker> | 44.99€ |
| Housing for heating mat | Heat resistant | 3D-printed funnel (STEP file) and high pressure laminate plates |  |
| Standoffs for heating mat | Heat resistant | Turned aluminum rods |  |
| Humidity control | | | |
| Designation | feature | Source (link to product or file) | Price |
| Ultrasonic evaporators with control | One evaporator sufficient for volume of 0.5 m^3^ to reach an increase in humidity of 15% at 24°C | <https://www.amazon.de/-/en/Iegefirm-Ultrasonic-Evaporator-Accessories-Humidifier/dp/B0BYYDM33B> | 16.91€ |
| Ultrasonic evaporators without control | Spare parts | <https://www.amazon.de/-/en/Transducer-Atomizing-Ultrasonic-Humidifier-Accessories/dp/B07V72T1T9> | 6.99€ |
| 3d-printed siphon | Holds standard hard PET bottles that do not deform | 3D-printed (STEP file) |  |
| Lighting | | | |
| Designation | feature | Source (link to product or file) | Price |
| Power supply white LEDs growing chamber | Can power 44 white LEDs (4 in series, 11 strips in parallel) | <https://www.reichelt.com/de/en/shop/product/led-switching_power_supply_elg_60_w_12_v_dc_5_a-185913> | 42.90€ |
| Power supply red LEDs growing chamber | Can power 20 red LEDs (4 in series, 5 strips in parallel) | <https://www.conrad.de/de/p/mw-mean-well-lpf-60d-12-led-treiber-led-trafo-konstantspannung-konstantstrom-60-w-5-a-7-2-12-v-dc-dimmbar-pfc-schal-1297240.html?refresh=true> | 36.27€ |
| Spacer for PCBs | Holds LED strips above acrylic lid | 3D-printed (STEP file) |  |
| PCB design | Homogenous lighting within the box, adjustable distances between PCBs, LEDs evenly distributed  Two edge strips with two rows of LEDs | wiring diagram (Supplementary material 3) |  |
| White LEDS | Frequency curve as similar as possible to sunlight | <https://lumileds.com/products/high-power-leds/luxeon-hl2x/> | 0.86€ /pc |
| Red LEDS | Important for photomorphogenesis | <https://ams-osram.com/de/products/leds/color-leds/osram-led-engin-luxigen-lz1-00r202> | 3.68€ /pc |
| Horizontal 70 mm in bore extension | | | |
| Designation | feature | Source (link to product or file) | Price |
| Insert fan | High pressure, high flow | <https://www.conrad.de/de/p/ebm-papst-624-2h3p-axialluefter-24-v-dc-67-m-h-l-x-b-x-h-60-x-60-x-25-mm-1926357.html> | 27.72€ |
| Acrylic insert tube | Fits within bore, thin walled, leaving space for plants | <https://www.erson.pl/rura-przezroczysta-pleksi-70mm66mm-1m-p-125.html> | 17.30€ |
| Power supply insert | Powers 15 LEDs in series, 3 strips | <https://www.mouser.de/ProductDetail/MEAN-WELL/NPF-90D-42?qs=XCwcpc%2FLnasMcw6u89ZHHA%3D%3D> | 26.04€ |
| Flexible tube | Improve handling and positioning of extension | <https://www.voelkner.de/products/9822941/Absaug-u.Geblaeseschlauch-PROTAPE-PUR-301-AS-ID-80mm-AD-88mm-4mm-L.5m-NORRES.html> | 18.00€ /m |
| Connection tube | PVC tube to extend inside the scanner and not to cause artifacts | <https://www.landefeld.de/artikel/de/leichter-saug-schlauch-pvc-superflex-grau-60mm/VU%2060%20FLEX> | 17.07€ /m |
| Insert extension | Control light in-bore | 3D-printed (STEP file) |  |
| Adapter: insert to connection tube | Connect insert to connection tube | 3D-printed (STEP file) |  |
| Adapter: connection tube to flexible tube | Connect connection tube to flexible tube | 3D-printed (STEP file) |  |
| Adapter: chamber to flexible tube | Quick removal and installation of flexible tube to move box freely | 3D-printed (STEP file) |  |
| PCB design for LEDs | Homogenous lighting within the insert | wiring diagram (Supplementary material 4) |  |


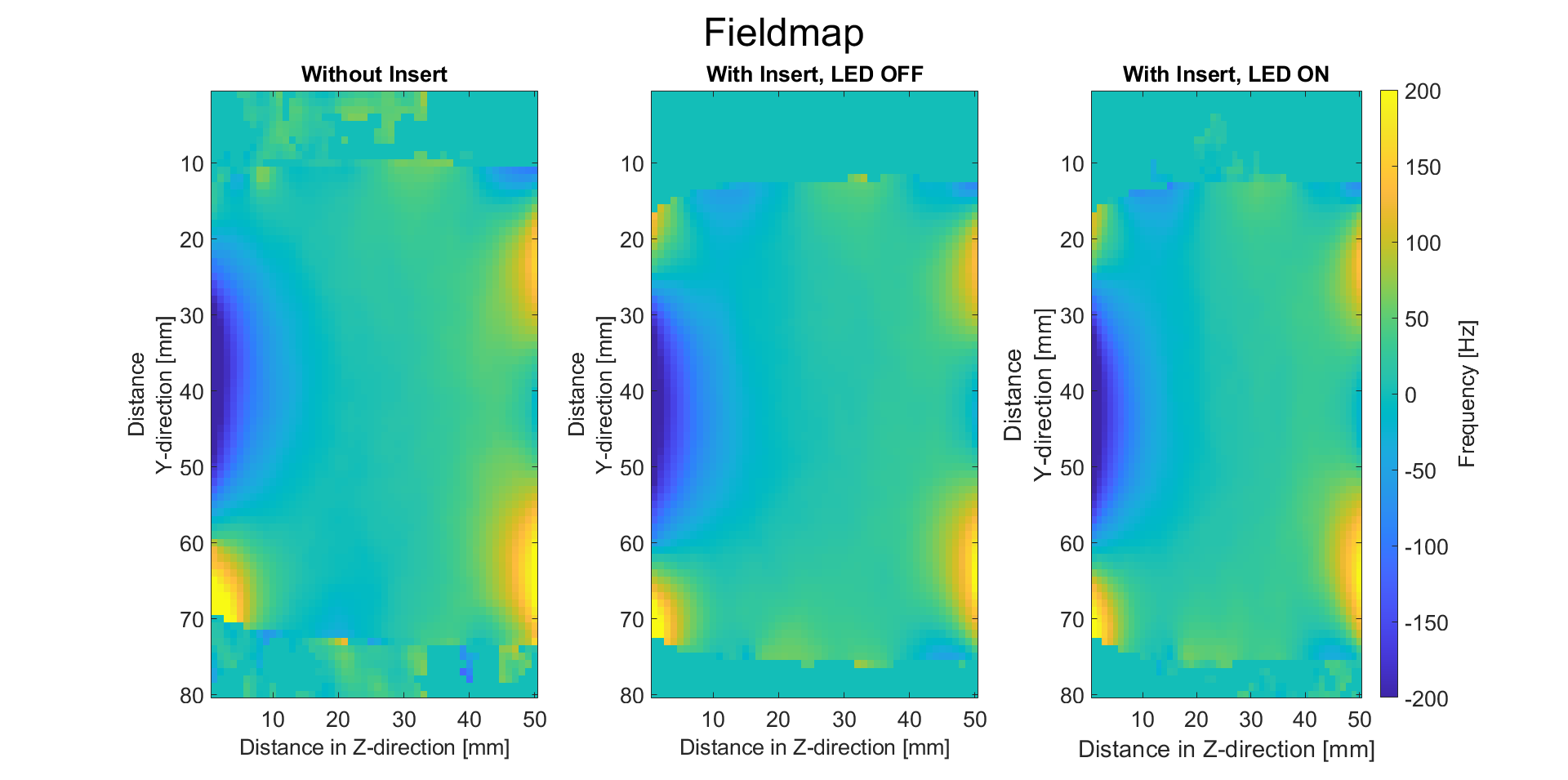


Figure S 1: Representative slice of a 3D B_0_ field map, measured without the insert (left) and with the insert installed with both the LEDs turned off (middle) and on (right). No significant differences in field homogeneity can be detected. Field maps were acquired with a double-echo FLASH sequence (TE = 2 ms and 5 ms) in 2:59 min per field map. Voxel size was 750x750x938 µm.

# Additional supplementary material

Supplementary material 1: The PDF “PCB-layout-control-system” contains the annotated circuit diagram of the main control system.

Supplementary material 2: The PDF “PCB-layout-adapter-dimming” contains the annotated circuit diagram of the dimming adapter.

Supplementary material 3: The PDF “PCB-layout-LED-strips-chamber” contains the annotated circuit diagram of the LED strips for the main chamber.

Supplementary material 4: The PDF “PCB-layout-LED-strips-extension” contains the annotated circuit diagram of the LED strips in the extension.

Further supplementary material: The folder 3d-files in the github repository contains .STP files of the main chamber, the insert, and the connectors.

## Software

Software is uploaded to Github and can be accessed from: <https://zenodo.org/records/18861390>
